# The Prevalence of Metabolic Syndrome and Health-Related Behavior Changes: The Korea National Health Examination Survey

**DOI:** 10.3390/healthcare8020134

**Published:** 2020-05-15

**Authors:** Eunshil Yim, Kyounga Lee, Ilsu Park, Sangjin Lee

**Affiliations:** 1 Department of Nursing, Daegu Health College, 15 Yeongsong-ro, Buk-gu, Daegu 41453, Korea; yim7604@dhc.ac.kr; 2 Medical Research Collaborating Center, Seoul National University Hospital, 101, Daehak-ro, Jongno-gu, Seoul 03080, Korea; 3 Department of Healthcare Management, Dong-eui University, 176 Eomgwangno, Busanjin-gu, Busan 47340, Korea; ispark@deu.ac.kr; 4 Ministry of Health and Welfare, 13, Doum 4-ro, Sejong-si 30113, Korea; sjlee0709@korea.kr

**Keywords:** metabolic syndrome, health behavior, smoking, alcohol intake, physical activity

## Abstract

This study was conducted to investigate the effect of health-related behavior changes on the prevalence of metabolic syndrome (MetS). This study utilized data from the Korea National Health Examination Survey of adults aged 40 or older who underwent health screening in 2011, 2013, and 2015. The prevalence of MetS was analyzed according to sex, age, income, residence location, and health-related behaviors by conducting multiple logistic regression analysis. For health-related behaviors, smoking, drinking, and physical activity were examined, and changes in health-related behaviors over five years from 2011 to 2015 were included in the analysis. The prevalence of MetS in Korea in 2015 was 31.7%. The prevalence showed statistically significant differences according to sex, age, income, location, and health-related behaviors. The prevalence was higher in men than in women and increased with aging. Regarding income, MetS prevalence was slightly higher in the middle-income groups compared with the lowest or the highest. Regarding location, MetS prevalence was lower in metropolitan areas compared to small- to medium-sized cities and farming/fishery rural areas. Regarding health-related behavior, MetS prevalence increased in the smoking, heavy drinking, and passive activity groups compared with the nonsmoking, moderate drinking, and active activity groups. Regarding health-related behavior change, MetS prevalence was higher by 22% in the short-term nonsmoking group (subjects who smoked in the past but not currently) compared to the continuous nonsmoking group. The risk for MetS also increased by 84.9% in the continuous heavy drinking group compared to the continuous moderate drinking group. Finally, the risk for MetS increased by 30.3% in the continuous passive physical activity group compared to the continuous active physical activity group. This study’s findings indicate the importance of maintaining healthy lifestyle habits to prevent MetS. In particular, the focus for change should be concentrated on short-term nonsmoking, continuous heavy drinking, and continuous passive physical activities to improve health-related behaviors.

## 1. Introduction

Metabolic syndrome (MetS) is conceptualized as a cluster of conditions with a high risk for cardiovascular disease and type 2 diabetes, and the presence of MetS is reported to increase the risk for these diseases [1]. The mechanism of the occurrence of MetS is not yet clear, but diverse personal and environmental factors (such as smoking, drinking, exercise habits, family history, and education) as well as genetics have been found to be involved [2,3,4,5,6]. According to a meta-analysis study on the relationship between smoking and MetS, there is a significant and positive relationship between active smoking and an increased risk of MetS [2]. Self-reports of moderate consumption of alcohol were associated with a reduction in the prevalence of MetS, serum lipid concentration, and waist circumference compared to those who reported heavy alcohol consumption [3,4]. Furthermore, decreased physical activity and an increase in the number of sleeping hours increased MetS prevalence [5]. In another study, the key determinants of MetS were exposure to sedentary lifestyles and obesity [6]. Hence, MetS and cardiac and cerebrovascular disease are believed to be preventable through health-related behaviors such as smoking cessation, moderate drinking, exercise and weight control, and the management of blood pressure, blood sugar, and cholesterol. The National Cholesterol Education Program—Adult Treatment Panel III (NCEP-ATP III) recommends aggressive therapeutic lifestyle changes, including diet, exercise, and education, as a primary strategy in managing patients with MetS [1,7,8]. 

There has been a significant increase in the prevalence of MetS throughout the world [7,9]. Asian countries have not escaped this trend. In Korea, MetS prevalence in adults has rapidly increased from 24.9% in 1998 to 31.3% in 2007, indicating that one out of three adults in Korea has MetS [10]. Currently, seven out of ten primary causes of death in Korea involve chronic degenerative diseases. Of those, cardiac and cerebrovascular diseases have become the primary causes of death, constituting as much as 25.8% of mortality cases [11]. Accordingly, the National Health Promotion Act was created in Korea for health promotion and disease prevention and the National Health Plan 2020 was established for the promotion of physical activity. A variety of policies were promoted to increase the urgency of improving health-related behaviors and reducing smoking, drinking, and obesity. One of these policies, already implemented, has been conducting health screening and lifestyle habit assessments at least biennially in citizens aged 40 or older to maintain and promote health, minimize economic loss, and reduce health insurance expenditures over a long term. Lifestyle interventions should be conducted at the national level to reduce the burden and consequences of metabolic syndrome [10]. 

However, it is not easy to modify lifestyle habits that have formed over an extended period of time, and even if a person begins to improve their health-related behaviors, maintaining these new behaviors is not easily done. Among the risk factors for MetS, smoking is a modifiable factor and most smokers attempt to quit smoking. However, less than 5% of those who attempt smoking cessation succeed in doing so over 12 months, and the success rate for those who reattempt to quit smoking after having previously failed to do so is under 20% [12]. 

Health-related behavior change should be maintained over a long period to achieve health benefits because the duration of such change has an impact on the risks for MetS and chronic illnesses [13]. Most previous studies, however, were cross-sectional in design, only investigating the relationship between health-related behavior performance and MetS at the time of study and did not consider the duration of maintaining these behaviors and the pattern of change. A few studies did examine the effects of lifestyle interventions over a six to twelve-month period, but the findings were inconsistent. The researchers pointed out that with a short-term intervention, it may be difficult to see clear effects and that additional research should be conducted to confirm whether the effects of lifestyle modification are retained following the intervention [14,15,16,17]. However, it is difficult to maintain healthy behaviors and individuals often revert to unhealthy habits, and therefore the pattern of change should be considered. Thus, the prevalence of MetS should be studied over time regarding health-related behavior changes within a subject and considering the duration of health-related behavior performance. The purposes of this study were to examine the pattern of within-subject changes in smoking, drinking, and physical activity and to identify influencing factors on the prevalence of MetS among health-related behavior changes.

## 2. Materials and Methods

### 2.1. Study Design

This research was a descriptive and retrospective cohort study aimed at investigating the effects of health-related behavior changes on the prevalence of MetS, using data from the Korea National Health Examination Survey of adults aged 40 or older who underwent general health screening performed by the Korea National Health Insurance Service in 2011, 2013, and 2015.

### 2.2. Data Collection and Ethical Considerations

Upon the approval of the K University Institutional Review Board (IRB No. E1507/001-003), an information disclosure request was made to the Korea National Health Insurance Service. Specifically, data on health screening, insurance eligibility, insurance premiums, and insurance claims were requested. Of the data received from the Korea National Health Insurance Service, those of a total of 578,416 adults aged 40 or older who underwent health screening in 2011, 2013, and 2015 were analyzed in this study.

### 2.3. Measuring Health-Related Behavior and Metabolic Syndrome

#### 2.3.1. General Characteristics

Sex was categorized into male and female, and age was grouped into intervals of 10 years. Residence location was classified as metropolitan area, small- to medium-sized city, and farming/fishery rural area. Income level was sorted into quintiles, with a higher quintile ranking indicating a higher income level.

#### 2.3.2. Health-Related Behavior 

Health-related behavior was defined for each of the items regarding smoking, drinking, and physical activity in the general health screening survey. Smoking was classified into groups of smokers and nonsmokers, which were further defined as current smokers and current nonsmokers including former smokers, respectively, in accordance with the National Health Interview Survey of the US Centers for Disease Control (US CDC). Current smokers referred to those who had smoked over 100 cigarettes during their lifetime and who currently smoke, while nonsmokers referred to those who had never smoked or smoked under 100 cigarettes during their lifetime. Former smokers referred to those who had previously smoked over 100 cigarettes during their lifetime but who do not currently smoke [18]. Drinking was classified into groups of moderate drinkers and heavy drinkers. Heavy drinkers were defined as consuming, on average, more than seven glasses of any alcoholic beverage for men and five glasses for women at least twice per week. All others were classified into the moderate drinking group. These criteria are used in the Korea National Health and Nutritional Examination Survey (KNHANES) in accordance with the World Health Organization (WHO)’s criteria for high-risk alcohol consumption of 60 g per day for men and 40 g per day for women [19]. Physical activity was categorized as active and passive, based on the criteria used in the Global Physical Activity Questionnaire (GPAQ) [20]. Being active in physical activity refers to performing one or more activities from high-intensity activity, medium-intensity activity, and walking. The high-intensity activity group consisted of subjects who performed a vigorous workout 30 minutes for more than three times in the past week, and medium-intensity activity group consisted of those who performed a moderately intense workout 30 minutes for more than five times in the past week. The walking group consisted of subjects who walked 30 minutes for more than five times in the last week. 

#### 2.3.3. Health-Related Behavior Change

Changes in smoking behavior were classified as continuous nonsmoking, short-term nonsmoking, short-term smoking, and continuous smoking. The continuous nonsmoking group consisted of subjects who were nonsmokers in 2011, 2013, and 2015, while the continuous smoking group consisted of subjects who were current smokers throughout that period. The short-term nonsmoking group included subjects classified as nonsmokers in 2015 but as current smokers in 2011 and 2013. The short-term smoking group included subjects classified as current smokers in 2015 but as nonsmokers in 2011 and 2013. 

Changes in drinking behavior were classified as continuous moderate drinking, short-term moderate drinking, short-term heavy drinking, and continuous heavy drinking. The continuous moderate drinking group consisted of subjects classified as moderate drinkers in 2011, 2013, and 2015, while the continuous heavy drinking group consisted of subjects classified as heavy drinkers throughout. The short-term moderate drinking group consisted of subjects classified as moderate drinkers in 2015 but as heavy drinkers in 2011 and 2013. The short-term heavy drinking group consisted of subjects classified as heavy drinkers in 2015 but as moderate drinkers in 2011 and 2013. 

Changes in physical activity were categorized as continuous passive, short-term passive, short-term active, and continuous active. The continuous passive group consisted of subjects classified as passive in their physical activity in 2011, 2013, and 2015, while the continuous active group consisted of subjects classified as active throughout. The short-term passive group consisted of subjects classified as passive in 2015 but as active in 2011 and 2013, and the short-term active group consisted of subjects classified as active in 2015 but as passive in 2011 and 2013.

#### 2.3.4. Metabolic Syndrome

The diagnosis of MetS was based on the modified ATP III criteria 13 presented by the American Heart Association (AHA) and the US National Heart, Lung, and Blood Institute (NHLBI), in which racial differences are considered [21]. Considering the difference between Asians and Westerners in the distribution of waist circumference, this study used the Korean Society for the Study of Obesity (KOSSO) criteria for abdominal obesity in the Korean population, i.e., 90 cm for men and 85 cm for women [22]. Subjects who met three of the five items of abdominal obesity as determined by waist circumference, hypertriglyceridemia, low HDL cholesterol, high blood pressure, and high fasting glucose were diagnosed for MetS.

### 2.4. Statistical Analysis

Differences in MetS prevalence by sex, age, income, location, and health-related behaviors were analyzed by χ^2^ tests. Factors influencing MetS prevalence were identified by conducting multiple logistic regression analysis and examining odds ratios (ORs). All data analyses were performed using SPSS 24 version (IBM SPSS Statistics, Armonk, NY, USA).

## 3. Results

### 3.1. General Characteristics

The total number of study subjects was 578,416. Of those, 243,222 (42.0%) were male, and 335,194 (58.0%) were female (Table 1). Regarding age, there were 115,543 (20.0%) subjects in the group of 40–49, 229,769 (39.7%) in the group of 50–59, 153,016 (26.5%) in the group of 60–69, and 80,088 (13.8%) in the group of 70 or older. Regarding income, 176,782 (30.6%) were assigned to the fifth quintile group (the highest income level), which was the largest of the five quintile groups. Regarding location, 276,215 (47.8%) lived in a metropolitan area, 230,053 (39.8%) in a small- to medium-sized city, and 72,148 (12.5%) in a farming/fishery rural area. With respect to health-related behaviors, 496,860 (85.9%) were classified into the nonsmoking group and 81,556 (14.1%) into the smoking group. Regarding drinking, 514,111 (88.9%) were classified into the moderate drinking group and 64,305 (11.1%) into the heavy drinking group. Lastly, 338,302 (58.5%) were classified into the passive and 240,114 (41.5%) in the active physical activity group.

### 3.2. Changes in Metabolic Syndrome Prevalence

The prevalence of MetS increased from 28.5% in 2011 and 28.6% in 2013 to 31.7% in 2015 (Table 2). The prevalence showed statistically significant differences according to sex, age, income, location, and health-related behaviors (*p* < 0.001). MetS prevalence was higher in men than in women, and increased with increasing age, with approximately 20% in the group of 40–49 and over 40% in the group of 70 or older with a MetS diagnosis. Regarding income level, the prevalence was the lowest in the fifth quintile (the highest income level), and regarding location, it was the highest in farming/fishery rural areas and the lowest in metropolitan areas. With respect to health-related behaviors, the prevalence was higher in the smoking group, the heavy drinking group, and the passive group, compared to the nonsmoking group, the moderate drinking group, and the active group, respectively. The general characteristics according to the presence or absence of MetS was suggested as Supplemental Materials (Table S1).

### 3.3. Changes in Health-Related Behaviors

Regarding health-related behavior changes, the largest group with respect to smoking was the continuous nonsmoking group (457,133, 79.0%), followed by the continuous smoking group (63,012, 10.9%), the short-term nonsmoking group (39,727, 6.9%), and the short-term smoking group (18,544, 3.2%). With respect to drinking, the largest group was the continuous moderate drinking group (465,147, 80.4%), followed by the short-term moderate group (48,964, 8.5%), the short-term heavy drinking group (37,460, 6.5%), and the continuous heavy drinking group (26,845, 4.6%). With respect to physical activity, the largest group was the continuous passive group (183,138, 31.7%), followed by the short-term active group (161,729, 28.0%), the short-term passive group (155,164, 26.8%), and the continuous active group (78,385, 13.5%). 

There were differences in health-related behavior changes according to sex, age, income, and location (*p* < 0.001). Regarding sexual differences, 23.2% of men were classified into the continuous smoking group and the rate of women classified into the group was 2.0%. Additionally, 9.8% of men and 0.9% of women were in the continuous heavy drinking group, and 30.3% of men and 32.7% of women were in the continuous passive group. Hence, with respect to health-related behavior changes, women engaged in healthier behaviors regarding smoking and drinking, whereas men were engaged in healthier physical activity. Regarding age differences, smoking and drinking decreased and physical activity increased with increasing age. Regarding differences by income level, the second and third quintile groups showed the highest rates of continuous smoking, heavy drinking, and passivity in physical activity. Regarding differences by location, the rate of active physical activity was higher in urban areas compared to rural areas, which also corresponded to the rates of smoking and drinking. (Table 3). The general characteristics according to health-related behavior was suggested as Supplemental Materials (Table S2).

### 3.4. Health-Related Behavior Changes and Metabolic Syndrome Prevalence

The examination of MetS prevalence according to health-related behavior changes showed that regarding smoking, the prevalence was the highest in the short-term nonsmoking group (37.2%). Regarding drinking, the prevalence was the highest in the continuous heavy drinking group (40.8%), and regarding physical activity, it was the highest in the short-term passive group (32.9%) (Table 4). Of all health-related behavior groups, the continuous heavy drinking group showed the highest MetS prevalence (40.8%) and the continuous active group the lowest prevalence (29.4%).

### 3.5. The Factors Influencing the Risk of Metabolic Syndrome Prevalence 

To identify factors influencing the risk of MetS prevalence, multiple logistic regression analysis was performed with the presence or absence of MetS as the dependent variable. The sex, area, age, income level, the status of smoking/drinking/activity, and the presence or absence of MetS were determined in the year 2015 data. Health-related behavior change was determined using data from 2011, 2013, and 2015. The analysis results are presented in Table 5. Two regression models were constructed with subjects’ general characteristics controlled by including all the variables in each of the models. In Model 1, the current smoking, drinking, and physical activity status were added as independent variables, while in Model 2 the changes in smoking, drinking, and physical activity were included. The results of Model 1 showed that the risk for MetS was higher by 9.7% in the smoking group vs. the nonsmoking group, by 54.9% in the heavy drinking group vs. the moderate drinking group, and by 18.7% in the passive group vs. the active group. However, in Model 2, which included health-related behavior changes, it was found that the risk for MetS was the highest in the short-term nonsmoking group (by 22%, using the continuous nonsmoking group as the reference), followed by the short-term smoking group (by 11.6%) and the continuous smoking group (by 10.5%). Regarding changes in drinking behavior, the risk for MetS increased from the short-term moderate group (by 32.3%, with the continuous moderate drinking group as the reference) to the short-term heavy drinking group (by 48.3%) to the heavy drinking group (by 84.9%). Regarding changes in physical activity, the risk for MetS increased from the short-term active group (by 11.4%, with the continuous active group as the reference) to the short-term passive group (by 23.9%) to the continuous passive group (by 30.3%). In addition to health-related behavior changes, the following characteristics affected the prevalence of MetS: it was higher in women than in men, in small- to medium-sized cities or in farming/fishery rural areas than in metropolitan areas, in age groups of 50 or older than in the age group of 40–49, and in the fourth or lower-income quintile group than in the fifth quintile group.

## 4. Discussion

In this descriptive longitudinal study, observational analysis was performed to identify factors influencing the prevalence of MetS, using data from the Korea National Health Examination Survey of a total of 578,416 adults aged 40 or older. Smoking, drinking, and physical activity are modifiable health-related behaviors and they are very critical in the management of MetS. Although MetS develops gradually and over a long time, most studies have examined whether subjects performed health-related behaviors only at the time of data collection, not considering the duration over which they performed such behaviors. To overcome this limitation, the present study utilized data from the Korea National Health Examination Survey conducted periodically (i.e., every other year) to examine the effects of health-related behavior changes. However, because health-related behaviors change over time, data from the Korea National Health Examination Survey in 2011, 2013, and 2015 were utilized to investigate the effects of health-related behavior changes on MetS prevalence.

### 4.1. Metabolic Syndrome Prevalence

The prevalence of MetS in Korea was 28.5% in 2011, 28.6% in 2013, and 31.7% in 2015. According to the Korean Society of Cardiometabolic Syndrome, the 2015 MetS prevalence was 20.3% in adults aged 19 or older, 27.0% in adults aged 30 or older, and 37.7% in adults aged 65 or older [23]. Considering that this study’s subjects were aged 40 or older, this study’s findings on MetS prevalence appear representative. In a study based on the US National Health and Nutrition Examination Survey, MetS prevalence in 2014 in the US was also at a similar level (31.5%), but the age range of this study sample was 20 to 85 [24]. In contrast, the sample of this study included adults aged 40 or older, and given that MetS prevalence increases with age, the prevalence in the population of the same age range as in the US study is likely to be lower in Korea. The discrepancy is attributable to the differences in abdominal obesity criteria (the NCEP-ATP Ⅲ criterion of 80 cm vs. 85 cm in this study), genetic factors, and lifestyle habits [25]. 

MetS manifests differently depending on sex, age, and socioeconomic characteristics, and different studies have reported different findings. In this study, MetS prevalence was statistically significantly different according to sex, age, income, and location. The prevalence was higher in men than in women, and in older adults than in those who were younger [25]. These findings are congruous with the finding that abdominal obesity and blood pressure increase with aging. In addition, it is due to an increase in sedentary lifestyles, functional disabilities, and a decrease in physical activity with aging [26,27]. Regarding income, the highest quintile group showed the lowest prevalence, and regarding location, it was metropolitan areas that showed the lowest prevalence. These findings confirm an association between socioeconomic status and MetS prevalence, as shown by a previous study [26].

### 4.2. Association between Health-Related Behavior Changes and Metabolic Syndrome Prevalence

Health-related behaviors showed differences according to sex, age, income, and location. With respect to sex, women showed higher rates of continuous nonsmoking and moderate drinking. With respect to age, older age groups showed higher rates of continuous nonsmoking, moderate drinking, and physical activity. These findings suggest an association between maintaining healthy lifestyle habits and longevity. With respect to income, the rates of continuous smoking, heavy drinking, and passive physical activity were higher in the middle quintile groups (second through fourth) than in the lowest or highest quintile groups. This confirms the finding of various studies that a higher income is directly related to higher health status. Simultaneously, this suggests that the middle-income groups may neglect conducting a healthy lifestyle due to a lack of time, an information gap, and economic alienation, whereas the lowest income group is at the center of focus for various systems and can get assistance with healthy physical activity. 

Different studies have reported different findings regarding the relationships between MetS and smoking, drinking, and physical activity. In some studies, the relationships were statistically significant while in others they were not. However, in this study MetS prevalence was higher in the smoking group than in the nonsmoking group, in the heavy drinking group than in the moderate drinking group, and in the passive group than in the active group, demonstrating statistically significant relationships between MetS and smoking, drinking, and physical activity [5,28].

A close examination of health-related behavior changes showed that the rates of groups attempting to quit smoking and drink moderately are higher compared with those who started to smoke or drink heavily, and that the rate of those who proactively tried to increase physical activity was also high. These findings are interpreted as indicating that the number of individuals highly interested in health-related behaviors and trying to maintain their health is increasing. Regarding the prevalence of MetS according to the changes in health-related behaviors, all groups, except for the continuous nonsmoking, continuous moderate drinking, and continuous active groups, showed MetS prevalence above the mean. These findings demonstrate the importance of considering not only the current status of smoking, drinking, and physical activity, but also health-related behaviors in the past and how long a health-related behavior has been maintained. In particular, there has been a close association demonstrated between smoking and MetS as well as its components [29], and it should be noted that in this study, MetS prevalence was higher in the short-term nonsmoking group than in the continuous smoking group. This finding urges researchers to consider why smokers try to quit smoking. Considering that they are motivated to quit smoking due to health reasons, the short-term nonsmoking group (i.e., those who smoked in the past but not currently) should undoubtedly be a subject of focus and managed consistently, in addition to the continuous smoking group.

### 4.3. The Risk of Metabolic Syndrome Prevalence 

In the analysis based simply on the current smoking, drinking, and physical activity status as independent variables, the risk for MetS increased by 9.7% in the smoking group vs. the nonsmoking group, by 54.9% in the heavy drinking group vs. the moderate drinking group, and by 18.7% in the passive group vs. the active group. The greatest difference in the findings after health-related behavior changes were included in the analysis (Model 2) was that the risk of MetS prevalence was higher by 22% in the short-term nonsmoking group using the continuous nonsmoking group as the reference. In contrast, it was higher by 11.6% in the short-term smoking group and by 10.5% in the continuous smoking group, demonstrating that the risk is higher in the short-term nonsmoking group than it is in the continuous smoking group.

Numerous studies have reported that the risk for MetS increases in smokers compared to nonsmokers. In this study, however, the risk was found to be the highest in the group of former smokers who had currently quit smoking. Smokers who already had other health problems may have decided to quit smoking due to those health problems, which may have put them at an increased risk for MetS. An alternative interpretation of this finding is that weight gain occurred as a result of smoking cessation. The phenomenon of weight gain after smoking cession is well known. In comparison to those who continue smoking, weight, blood pressure, and cholesterol levels increase for individuals who quit smoking [30,31]. It has been argued that the health benefits due to smoking cessation may be lower than previously thought because weight gain elevates the risk for cardiovascular disease. However, a study reported that although weight initially increased with smoking cessation, the increase was attenuated over time, with no difference between former smokers and nonsmokers after five years [32]. Accordingly, more attention should be paid to the prevention of weight gain in the short-term nonsmoking group, and smokers attempting to quit smoking should be closely managed so that they do not return to smoking because of weight gain.

The risk for MetS increased by 84.9% in the continuous heavy drinking group compared with the continuous moderate drinking group, suggesting the need to avoid heavy drinking. The findings on the association between drinking alcohol and the prevalence of MetS and its components are inconsistent [33]. In this study, however, the risk for MetS associated with heavy drinking was higher than the risk associated with smoking or lack of physical activity, clearly showing the need for moderating drinking. In Korea, the rate of heavy drinking is very high and alcohol is the leading cause of the burden of disease [34]. Hence, an effort should be made to avoid heavy drinking to prevent MetS.

Regarding physical activity, MetS prevalence was higher in the passive groups than in the continuous active group, suggesting the need to increase physical activity. As the rate of physical activity is reported to be 38.7% in Korea while it is 50.3% in major OECD countries, this study’s findings demonstrate the need to develop realistic and practical programs to increase physical activity.

Lastly, the risk for MetS was higher in small- to medium-sized cities and rural areas than in metropolitan areas, in adults aged 50 or older than those in their 40s, and in lower-income quintile groups than in the highest. It is speculated that location and income level affected MetS prevalence because geographical and economic factors made it difficult to develop interest in disease prevention, improve health-related behaviors, conduct a healthy lifestyle, and have access to health screening. Specifically, according to Model 2, persistent changes in health-related behaviors had the greatest impact in the risk of MetS prevalence, except for age, which is not modifiable. Therefore, the effect on the risk of MetS prevalence would be great if focus is consistently directed toward the improvement of health-related behaviors in adults between the ages of 40 and 49 with the second quintile of income, who, as shown in Table 3, continuously smoke, drink heavily, and are passive in their physical activity.

Therefore, using the results of this study, it can suggest which group is effective to focus on to reduce the MetS prevalence. In addition, the importance of health-related behavior has been proved through this study; thus, these findings can be used as a basic resource for policy decision making for the practice of a healthy lifestyle.

Our study has the following limitations. First, this study sample did not include adults younger than 40 years old. Second, various other data, such as the volume of food consumption and genetic factors, were not taken into consideration. Third, the survey of health-related behaviors was based on self-reports and thus the data may have been influenced by recall and social desirability biases, thereby reducing accuracy. Finally, since this study focused on MetS prevalence, we did not analyze how health-related behavior change had an impact on MS incidence. Despite these limitations, the present study had significant strengths. First, our large sample of 578,416 citizens aged 40 or older was representative of the Korean population in those age groups. Second, this study is the first of its kind to include changes in health-related behaviors in addition to the behaviors at the time of data collection. Future studies should identify factors affecting the prevalence of MetS by including a wider variety of independent variables and considering the duration and absolute amount of health-related behavior performance over a longer term. In addition, it is necessary to analyze whether health-related behavior change affects not only MetS prevalence but also MetS incidence. 

## 5. Conclusions

This study was conducted to investigate the effect of health-related behavior change on the prevalence of MetS. Study findings showed that MetS prevalence was associated not only with socioeconomic factors but also with health-related behavior and change in the behavior. The prevalence increased in the smoking, heavy drinking, and passive groups compared with the nonsmoking, moderate drinking, and active groups, respectively. Regarding smoking, MetS prevalence was much higher in the short-term nonsmoking group (subjects who smoked in the past but not currently) than in the continuous smoking group. Accordingly, more attention should be paid to the health of smokers trying to quit smoking. In addition, the risk for MetS increased in heavy drinkers compared to moderate drinkers, suggesting the need to avoid heavy drinking. To summarize, the present study demonstrated that to prevent MetS, it is crucial to maintain healthy lifestyle habits.

## Figures and Tables

**Table 1:** General characteristics.

Categories			*n*	%
Total			578,416	100.0
Social Economic	Sex	Male	243,222	42.0
		Female	335,194	58.0
	Age	40–49	115,543	20.0
		50–59	229,769	39.7
		60–69	153,016	26.5
		≥70	80,088	13.8
	Income	1st (the lowest)	96,228	16.6
		2nd	82,501	14.3
		3rd	97,137	16.8
		4th	125,768	21.7
		5th (the highest)	176,782	30.6
	Location	Metropolitan area	276,215	47.8
		Small- to medium-sized cities	230,053	39.8
		Farming/fishery rural	72,148	12.5
Health-Related Behavior	Smoking	Nonsmoking	496,860	85.9
		Smoking	81,556	14.1
	Drinking	Moderate	514,111	88.9
		Heavy	64,305	11.1
	Physical activity	Passive	338,302	58.5
		Active	240,114	41.5

**Table 2:** Changes of metabolic syndrome prevalence.

Categories		2011				2013				2015			
		Normal		MetS		Normal		MetS		Normal		MetS	
		*n*	%	*n*	%	*n*	%	*n*	%	*n*	%	*n*	%
Total		413,480	71.5	164,936	28.5	413,079	71.4	165,337	28.6	394,967	68.3	183,449	31.7
Sex	Male	165,137	67.9	78,085	32.1	166,907	68.6	76,315	31.4	158,337	65.1	84,885	34.9
	Female	248,343	74.1	86,851	25.9	246,172	73.4	89,022	26.6	236,630	70.6	98,564	29.4
	χ^2^(*p*)	2652.629(<0.001)				1602.987(<0.001)				1965.273(<0.001)			
Age	40–49	167,810	80.9	39,596	19.1	127,035	81.6	28,642	18.4	90,919	78.7	24,624	21.3
	50–59	144,630	71.2	58,398	28.8	164,291	73.4	59,517	26.6	165,057	71.8	64,712	28.2
	60–69	75,818	61.7	47,099	38.3	85,626	63.4	49,606	36.6	94,857	62.0	58,159	38.0
	≥70	25,222	56.0	19,843	44.0	36,127	56.8	27,572	43.2	44,134	55.1	35,954	44.9
	χ^2^(*p*)	20160.172(<0.001)				19,524.344(<0.001)				16,332.791(<0.001)			
Income	1st	64,768	70.8	26,690	29.2	67,471	71.0	27,552	29.0	65,497	68.1	30,731	31.9
	2nd	59,963	71.7	23,650	28.3	58,114	71.8	22,782	28.2	56,757	68.8	25,744	31.2
	3rd	72,478	70.9	29,726	29.1	71,186	71.0	29,057	29.0	65,924	67.9	31,213	32.1
	4th	90,504	70.8	37,329	29.2	89,131	70.6	37,204	29.4	84,924	67.5	40,844	32.5
	5th	125,767	72.6	47,541	27.4	127,177	72.3	48,742	27.7	121,865	68.9	54,917	31.1
	χ^2^(*p*)	167.822(<0.001)				135.421(<0.001)				88.037(<0.001)			
Location	Metropolitan	201,625	73.0	74,590	27.0	200,397	72.6	75,818	27.4	191,999	69.5	84,216	30.5
	Cities	163,145	70.9	66,908	29.1	163,726	71.2	66,327	28.8	156,277	67.9	73,776	32.1
	Rural	48,710	67.5	23,438	32.5	48,956	67.9	23,192	32.1	46,691	64.7	25,457	35.3
	χ^2^(*p*)	903.877(<0.001)				629.403(<0.001)				629.381(<0.001)			
Smoking	Nonsmoking	341,941	72.0	133,245	28.0	346,140	71.8	136,162	28.2	341,310	68.7	155,550	31.3
	Smoking	71,539	69.3	31,691	30.7	66,939	69.6	29,175	30.4	53,657	65.8	27,899	34.2
	χ^2^(*p*)	294.113(<0.001)				176.929(<0.001)				272.387(<0.001)			
Drinking	Moderate	366,894	72.4	139,947	27.6	368,761	72.1	142,351	27.9	355,342	69.1	158,769	30.9
	Heavy	46,586	65.1	24,989	34.9	44,318	65.8	22,986	34.2	39,625	61.6	24,680	38.4
	χ^2^(*p*)	1640.276(<0.001)				1156.778(<0.001)				1483.481(<0.001)			
Physical Activity	Passive	255,738	71.1	104,152	28.9	247,393	70.7	102,523	29.3	227,167	67.1	111,135	32.9
	Active	157,742	72.2	60,784	27.8	165,686	72.5	62,814	27.5	167,800	69.9	72,314	30.1
	χ^2^(*p*)	84.345(<0.001)				221.745(<0.001)				484.817(<0.001)			

MetS: Metabolic Syndrome.

**Table 3:** Trend of changes in health-related behavior change by general characteristics.

**Smoking**		**Continuous Nonsmoking**		**Short-Term Nonsmoking**		**Short-Term Smoking**		**Continuous Smoking**		**χ^2^ (*p*)**
		**n**	**%**	**n**	**%**	**n**	**%**	**n**	**%**	
Total		457,133	79.0	39,727	6.9	18,544	3.2	63,012	10.9	
Sex	Male	140,397	57.7	31,925	13.1	14,444	5.9	56,456	23.2	116,272.686 (<0.001)
	Female	316,736	94.5	7802	2.3	4100	1.2	6556	2.0	
Age	40–49	83,155	72.0	8646	7.5	5069	4.4	18,673	16.2	10,819.038 (<0.001)
	50–59	176,996	77.0	16,778	7.3	8171	3.6	27,824	12.1	
	60–69	126,100	82.4	10,222	6.7	3944	2.6	12,750	8.3	
	≥70	70,882	88.5	4081	5.1	1360	1.7	3765	4.7	
Income	1st	75,379	78.3	6767	7.0	3311	3.4	10,771	11.2	2231.495 (<0.001)
	2nd	62,150	75.3	6487	7.9	3062	3.7	10,802	13.1	
	3rd	74,379	76.6	7282	7.5	3427	3.5	12,049	12.4	
	4th	99,849	79.4	8763	7.0	3748	3.0	13,408	10.7	
	5th	145,376	82.2	10,428	5.9	4996	2.8	15,982	9.0	
Location	Metropolitan	219,386	79.4	18,380	6.7	8662	3.1	29,787	10.8	68.201 (<0.001)
	Cities	181,009	78.7	16,096	7.0	7556	3.3	25,392	11.0	
	Rural	56,738	78.6	5251	7.3	2326	3.2	7833	10.9	
**Drinking**		**Continuous Moderate**		**Short-Term Moderate**		**Short-Term Heavy**		**Continuous Heavy**		**χ^2^ (*p*)**
		***n***	**%**	***n***	**%**	***n***	**%**	***n***	**%**	
Total		465,147	80.4	48,964	8.5	37,460	6.5	26,845	4.6	
Sex	Male	152,110	62.5	37,409	15.4	29,872	12.3	23,831	9.8	86,283.507 (<0.001)
	Female	313,037	93.4	11,555	3.4	7588	2.3	3014	0.9	
Age	40–49	83,584	72.3	11,722	10.1	11,048	9.6	9189	8.0	17,400.494 (<0.001)
	50–59	177,400	77.2	22,382	9.7	17,290	7.5	12,697	5.5	
	60–69	130,051	85.0	11,378	7.4	7341	4.8	4246	2.8	
	≥70	74,112	92.5	3482	4.3	1781	2.2	713	0.9	
Income	1st	78,077	81.1	8120	8.4	5976	6.2	4055	4.2	817.730 (<0.001)
	2nd	64,445	78.1	8022	9.7	5958	7.2	4076	4.9	
	3rd	76,675	78.9	9020	9.3	6,746	6.9	4696	4.8	
	4th	101,285	80.5	10,622	8.4	8165	6.5	5696	4.5	
	5th	144,665	81.8	13,180	7.5	10,615	6.0	8322	4.7	
Location	Metropolitan	222,263	80.5	23,249	8.4	17,848	6.5	12,855	4.7	10.592 (<0.001)
	Cities	185,039	80.4	19,386	8.4	14,965	6.5	10,663	4.6	
	Rural	57,845	80.2	6329	8.8	4647	6.4	3327	4.6	
**Physical activity**		**Continuous Passive**		**Short-Term Passive**		**Short-Term Active**		**Continuous Active**		**χ^2^ (*p*)**
		***n***	**%**	***n***	**%**	***n***	**%**	***n***	**%**	
Total		183,138	31.7	155,164	26.8	161,729	28.0	78,385	13.5	
Sex	Male	73,575	30.3	63,888	26.3	68,195	28.0	37,564	15.4	1423.333 (<0.001)
	Female	109,563	32.7	91,276	27.2	93,534	27.9	40,821	12.2	
Age	40–49	43,031	37.2	29,130	25.2	30,732	26.6	12,650	10.9	5346.539 (<0.001)
	50–59	76,354	33.2	61,510	26.8	63,928	27.8	27,977	12.2	
	60–69	40,600	26.5	41,890	27.4	45,353	29.6	25,173	16.5	
	≥70	23,153	28.9	22,634	28.3	21,716	27.1	12,585	15.7	
Income	1st	30,303	31.5	26,350	27.4	27,222	28.3	12,353	12.8	882.334 (<0.001)
	2nd	26,534	32.2	22,708	27.5	23,296	28.2	9963	12.1	
	3rd	31,404	32.3	26,652	27.4	27,065	27.9	12,016	12.4	
	4th	40,038	31.8	33,663	26.8	35,277	28.0	16,790	13.3	
	5th	54,859	31.0	45,791	25.9	48,869	27.6	27,263	15.4	
Location	Metropolitan	79,963	28.9	74,004	26.8	79,786	28.9	42,462	15.4	3588.688 (<0.001)
	Cities	76,431	33.2	60,924	26.5	63,354	27.5	29,344	12.8	
	Rural	26,744	37.1	20,236	28.0	18,589	25.8	6579	9.1	

**Table 4:** Health-related behavior change and metabolic syndrome prevalence.

Categories		Year			Normal		MetS	
		2011	2013	2015	*n*	%	*n*	%
Smoking	Nonsmoking	×	×	×	316,344	69.2	140,789	30.8
	Short-term nonsmoking	○	×	×	24,966	62.8	14,761	37.2
		×	○	×				
		○	○	×				
	Short-term smoking	×	×	○	12,268	66.2	6,276	33.8
		○	×	○				
		×	○	○				
	Smoking	○	○	○	41,389	65.7	21,623	34.3
	χ^2^ (*p*)				956.068 (<0.001)			
Drinking	Moderate	×	×	×	323,738	69.6	141,409	30.4
	Short-term moderate	○	×	×	31,604	64.5	17,360	35.5
		×	○	×				
		○	○	×				
	Short-term heavy	×	×	○	23,724	63.3	13,736	36.7
		○	×	○				
		×	○	○				
	Heavy drinking	○	○	○	15,901	59.2	10,944	40.8
	χ^2^ (*p*)				2127.236 (<0.001)			
Physical Activity	Passive	×	×	×	123,011	67.2	60,127	32.8
	Short-term passive	○	×	×	104,156	67.1	51,008	32.9
		×	○	×				
		○	○	×				
	Short-term active	×	×	○	112,426	69.5	49,303	30.5
		○	×	○				
		×	○	○				
	Active	○	○	○	55,374	70.6	23,011	29.4
	χ^2^ (*p*)				515.936 (<0.001)			

×: Nonsmoking, moderated drinking, passive in physical activity; ○: Smoking, heavy drinking, active in physical activity; MetS: Metabolic Syndrome.

**Table 5:** Multiple logistic regression for metabolic syndrome prevalence.

Variable		Reference Value	Model 1				Model 2			
			OR	95% CI		*p*	OR	95% CI		*p*
Sex	Female	Male	1.172	1.157	1.187	<0.001	1.097	1.082	1.111	<0.001
Area	Farming/fishery rural area	Metropolitan	1.494	1.468	1.519	<0.001	1.075	1.062	1.088	<0.001
	Small- to medium-sized cities		2.444	2.401	2.488	<0.001	1.112	1.092	1.132	<0.001
Age group	50–59	40–49	3.345	3.278	3.414	<0.001	1.505	1.480	1.531	<0.001
	60–69		1.103	1.084	1.122	<0.001	2.511	2.466	2.556	<0.001
	≥70		1.096	1.076	1.117	<0.001	3.481	3.410	3.553	<0.001
Income Level	1st (the lowest)	5th (the highest)	1.112	1.093	1.132	<0.001	1.094	1.075	1.113	<0.001
	2nd		1.085	1.068	1.102	<0.001	1.084	1.064	1.104	<0.001
	3rd		1.077	1.064	1.091	<0.001	1.102	1.083	1.121	<0.001
	4th		1.121	1.101	1.141	<0.001	1.078	1.061	1.095	<0.001
Smoking	Smoking	Nonsmoking	1.097	1.078	1.116	<0.001				
Drinking	Heavy	Moderate	1.549	1.521	1.579	<0.001				
Activity	Passive	Active	1.187	1.173	1.200	<0.001				
Smoking	Short-term nonsmoking	Nonsmoking					1.220	1.192	1.248	<0.001
	Short-term smoking						1.116	1.080	1.152	<0.001
	Smoking						1.105	1.083	1.127	<0.001
Drinking	Short-term moderate	Moderate					1.323	1.295	1.351	<0.001
	Short-term heavy						1.483	1.449	1.518	<0.001
	Heavy drinking						1.849	1.799	1.900	<0.001
Physical Activity	Passive	Active					1.303	1.279	1.328	<0.001
	Short-term passive						1.239	1.216	1.263	<0.001
	Short-term active						1.114	1.093	1.135	<0.001

OR: odds ratio; 95% CI: 95% confidence interval.

## Supplementary Materials

The following are available online at https://www.mdpi.com/2227-9032/8/2/134/s1, Table S1: The general characteristics according to the presence or absence of metabolic syndrome, Table S2: The general characteristics according to health-related behavior.
