# Supplementary material for: The Prevalence of Metabolic Syndrome and Health-Related Behavior Changes: The Korea National Health Examination Survey
Source: Healthcare (Basel). 2020 May 15;8(2):134. doi: 10.3390/healthcare8020134 (PMC7348718; doi:10.3390/healthcare8020134)
Supplement: Supplementary file 1 [file healthcare-08-00134-s001.pdf]

Table S1. The general characteristics according to the presence or absence of metabolic syndrome.

Categories		2011				2013				2015			
		**Normal**		**MetS**		**Normal**		**MetS**		**Normal**		**MetS**	
		n	%	*n*	%	*n*	%	*n*	%	*n*	%	*n*	%
**Total**		413,480	100.0	164,936	100.0	413,079	100.0	165,337	100.0	394,967	100.0	183,449	100.0
Sex	Male	165,137	39.9	78,085	47.3	166,907	40.4	76,315	46.2	158,337	40.1	84,885	46.3
	Female	248,343	60.1	86,851	52.7	246,172	59.6	89,022	53.8	236,630	59.9	98,564	53.7
Age	40–49	167,810	40.6	39,596	24.0	127,035	30.8	28,642	17.3	90,919	23.0	24,624	13.4
	50–59	144,630	35.0	58,398	35.4	164,291	39.8	59,517	36.0	165,057	41.8	64,712	35.3
	60–69	75,818	18.3	47,099	28.6	85,626	20.7	49,606	30.0	94,857	24.0	58,159	31.7
	≥70	25,222	6.1	19,843	12.0	36,127	8.7	27,572	16.7	44,134	11.2	35,954	19.6
Income	1st	64,768	15.7	26,690	16.2	67,471	16.3	27,552	16.7	65,497	16.6	30,731	16.8
	2nd	59,963	14.5	23,650	14.3	58,114	14.1	22,782	13.8	56,757	14.4	25,744	14.0
	3rd	72,478	17.5	29,726	18.0	71,186	17.2	29,057	17.6	65,924	16.7	31,213	17.0
	4th	90,504	21.9	37,329	22.6	89,131	21.6	37,204	22.5	84,924	21.5	40,844	22.3
	5th	125,767	30.4	47,541	28.8	127,177	30.8	48,742	29.5	121,865	30.9	54,917	29.9
Location	Metropolitan	201,625	48.8	74,590	45.2	200,397	48.5	75,818	45.9	191,999	48.6	84,216	45.9
	Cities	163,145	39.5	66,908	40.6	163,726	39.6	66,327	40.1	156,277	39.6	73,776	40.2
	Rural	48,710	11.8	23,438	14.2	48,956	11.9	23,192	14.0	46,691	11.8	25,457	13.9
Smoking	Non-Smoking	341,941	82.7	133,245	80.8	346,140	83.8	136,162	82.4	341,310	86.4	155,550	84.8
	Smoking	71,539	17.3	31,691	19.2	66,939	16.2	29,175	17.6	53,657	13.6	27,899	15.2
Drinking	Moderate	366,894	88.7	139,947	84.8	368,761	89.3	142,351	86.1	355,342	90.0	158,769	86.5
	Heavy	46,586	11.3	24,989	15.2	44,318	10.7	22,986	13.9	39,625	10.0	24,680	13.5
Physical Activity	Passive	255,738	61.9	104,152	63.1	247,393	59.9	102,523	62.0	227,167	57.5	111,135	60.6
	Active	157,742	38.1	60,784	36.9	165,686	40.1	62,814	38.0	167,800	42.5	72,314	39.4

Table S2. The general characteristics according to health-related behavior.

Smoking		Continuous Non-smoking		Short-term Non-smoking		Short-term Smoking		Continuous Smoking	
		n	%	*n*	%	*n*	%	*n*	%
**Total**		457,133	100.0	39,727	100.0	18,544	100.0	63,012	100.0
Sex	Male	140,397	30.7	31,925	80.4	14,444	77.9	56,456	89.6
	Female	316,736	69.3	7802	19.6	4100	22.1	6,556	10.4
Age	40–49	83,155	18.2	8,646	21.8	5069	27.3	18,673	29.6
	50–59	176,996	38.7	16,778	42.2	8171	44.1	27,824	44.2
	60–69	126,100	27.6	10,222	25.7	3944	21.3	12,750	20.2
	≥70	70,882	15.5	4081	10.3	1360	7.3	3765	6.0
Income	1st	75,379	16.5	6767	17.0	3311	17.9	10,771	17.1
	2nd	62,150	13.6	6487	16.3	3062	16.5	10,802	17.1
	3rd	74,379	16.3	7282	18.3	3427	18.5	12,049	19.1
	4th	99,849	21.8	8763	22.1	3748	20.2	13,408	21.3
	5th	145,376	31.8	10,428	26.2	4996	26.9	15,982	25.4
Location	Metropolitan	219,386	48.0	18,380	46.3	8662	46.7	29,787	47.3
	Cities	181,009	39.6	16,096	40.5	7556	40.7	25,392	40.3
	Rural	56,738	12.4	5251	13.2	2326	12.5	7833	12.4
**Drinking**		**Continuous Moderate**		**Short-term Moderate**		**Short-term Heavy**		**Continuous Heavy**	
		n	%	*n*	%	*n*	%	*n*	%
Total		465,147	100.0	48,964	100.0	37,460	100.0	26,845	100.0
Sex	Male	152,110	32.7	37,409	76.4	29,872	79.7	23,831	88.8
	Female	313,037	67.3	11,555	23.6	7588	20.3	3014	11.2
Age	40–49	83,584	18.0	11,722	23.9	11,048	29.5	9189	34.2
	50–59	177,400	38.1	22,382	45.7	17,290	46.2	12,697	47.3
	60–69	130,051	28.0	11,378	23.2	7341	19.6	4246	15.8
	≥70	74,112	15.9	3482	7.1	1781	4.8	713	2.7
Income	1st	78,077	16.8	8120	16.6	5976	16.0	4055	15.1
	2nd	64,445	13.9	8022	16.4	5958	15.9	4076	15.2
	3rd	76,675	16.5	9020	18.4	6746	18.0	4696	17.5
	4th	101,285	21.8	10,622	21.7	8165	21.8	5696	21.2
	5th	144,665	31.1	13,180	26.9	10,615	28.3	8322	31.0
Location	Metropolitan	222,263	47.8	23,249	47.5	17,848	47.6	12,855	47.9
	Cities	185,039	39.8	19,386	39.6	14,965	39.9	10,663	39.7
	Rural	57,845	12.4	6329	12.9	4647	12.4	3327	12.4
**Physical Activity**		**Continuous Passive**		**Short-term Passive**		**Short-term Active**		**Continuous Active**	
		n	%	*n*	%	*n*	%	*n*	%
Total		183,138	100.0	155,164	100.0	161,729	100.0	78,385	100.0
Sex	Male	73,575	40.2	63,888	41.2	68,195	42.2	37,564	47.9
	Female	109,563	59.8	91,276	58.8	93,534	57.8	40,821	52.1
Age	40–49	43,031	23.5	29,130	18.8	30,732	19.0	12,650	16.1
	50–59	76,354	41.7	61,510	39.6	63,928	39.5	27,977	35.7
	60–69	40,600	22.2	41,890	27.0	45,353	28.0	25,173	32.1
	≥70	23,153	12.6	22,634	14.6	21,716	13.4	12,585	16.1
Income	1st	30,303	16.5	26,350	17.0	27,222	16.8	12,353	15.8
	2nd	26,534	14.5	22,708	14.6	23,296	14.4	9963	12.7
	3rd	31,404	17.1	26,652	17.2	27,065	16.7	12,016	15.3

	4th	40,038	21.9	33,663	21.7	35,277	21.8	16,790	21.4
	5th	54,859	30.0	45,791	29.5	48,869	30.2	27,263	34.8
Location	Metropolitan	79,963	43.7	74,004	47.7	79,786	49.3	42,462	54.2
	Cities	76,431	41.7	60,924	39.3	63,354	39.2	29,344	37.4
	Rural	26,744	14.6	20,236	13.0	18,589	11.5	6579	8.4
